# Supplementary figures and images for: Spatial analysis of COVID-19 spread in Iran: Insights into geographical and structural transmission determinants at a province level
Source: PLoS Negl Trop Dis. 2020 Nov 18;14(11):e0008875. doi: 10.1371/journal.pntd.0008875 (PMC7710062; doi:10.1371/journal.pntd.0008875)

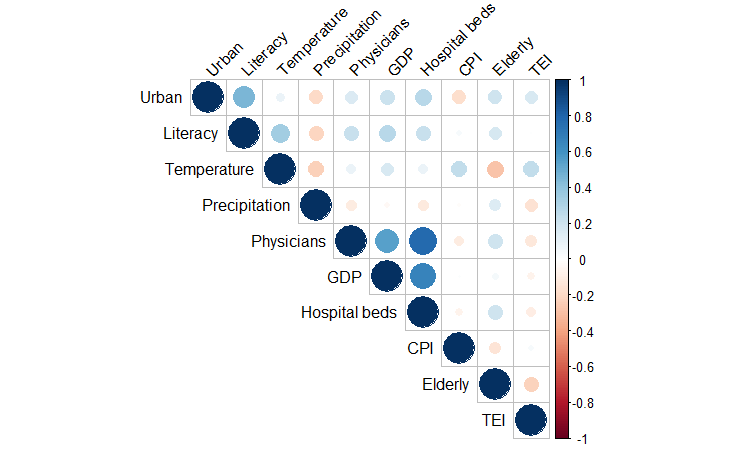

Supplement: S1 Fig — (PNG) [file pntd.0008875.s001.png]
