# Supplementary material for: Spatial analysis of COVID-19 spread in Iran: Insights into geographical and structural transmission determinants at a province level
Source: PLoS Negl Trop Dis. 2020 Nov 18;14(11):e0008875. doi: 10.1371/journal.pntd.0008875 (PMC7710062; doi:10.1371/journal.pntd.0008875)
Supplement: Attachment — Submitted filename: Review of PNTD.docx [file pntd.0008875.s002.docx]

Review of PNTD-D-20-00725: “Spatial analysis of COVID-19 spread in Iran: Insights into geographical and structural transmission determinants at a province level”

This manuscript is a regression analysis of COVID-19 patterns in Iran, performed at a provincial level, and taking into account the spatial structure of the variables used. As such, I found it interesting and well performed (however, I am no expert in public health). I have a few minor comments, but a more substantial one: this is a correlative study, but in the discussion, the language too often suggests an understanding of causation for which there is no evidence. Although this needs to be corrected, it is easy to rewrite the discussion using more careful wording.

Some details.

Lines 64-65. What is the meaning of “…its economic situation … is among the highest”? A situation cannot be high or low. Please rewrite.

Line 97. How was the data “standardized”? There are many ways of standardizing a variable. Be explicit.

Line 120. I believe they actually performed bootstrapping on the data, rather than “…Monte Carlo simulations”, for which the Moran paper cited is NOT a right reference. Correct this and be careful with the references.

Lines 136-137. A reference is required here. It is not clear to every reader what a “Lagrange Multiplier statistic” is.

Lines 234-235. You just showed statistical associations. Whether this shows or not what is needed to “model the disease adequately” is a different matter. The discussion needs a more careful language. For instance, in Line 237, what would be the role of variables in a mechanistic model is not established. Or (Line 265), that literacy is a “protective effect.” You just showed that some variables covary, not that one factors “protects” another.
