# Supplementary material for: Spatial analysis of COVID-19 spread in Iran: Insights into geographical and structural transmission determinants at a province level
Source: PLoS Negl Trop Dis. 2020 Nov 18;14(11):e0008875. doi: 10.1371/journal.pntd.0008875 (PMC7710062; doi:10.1371/journal.pntd.0008875)
Supplement: Attachment — Submitted filename: Answer_Reviewers_23072020.docx [file pntd.0008875.s003.docx]

July 23^rd^, 2020

**Dr. Townsend Peterson**

**Guest Editor**

**PLOS Neglected Tropical Diseases**

.

On behalf of my co-authors and myself I am re-submitting the manuscript entitled “Spatial analysis of COVID-19 spread in Iran: Insights into geographical and structural transmission determinants at a province level”. We acknowledge that the manuscript greatly improved when taking into account the comments of the reviewers. We send the corrected manuscript and the same manuscript with changes highlighted. At the bottom of this letter we include the comments by the reviewers along with our response, with the location of the changes when appropriate.

We appreciate the opportunity of considering our work,

Sincerely yours,

Ricardo Ramírez-Aldana, Juan Carlos Gomez-Verjan, Omar Yaxmehen Bello-Chavolla

Correspondence

Ricardo Ramírez-Aldana

Research Division

Instituto Nacional de Geriatría

Periférico Sur No. 2767

Col. San Jerónimo Lídice,

Del. La Magdalena Contreras, D.F. 10200

ricardoramirezaldana@gmail.com

I secured three reviews, each from an individual familiar with spatial statistics and/or spatial epidemiology. All three generally liked the analysis, but reviewers 1 and 2 had some rather serious concerns either about the methods, or about the description of the methods. I would like to see the authors rework their methods description rather deeply, and I will plan to have those two reviewers take a second look at the revised manuscript to assure that they are comfortable with the new descriptions.

**We have responded to all reviewers’ comments, particularly those related with the methods, in terms of what methods were applied and how they are explained in the manuscript. We also show to the reviewers additional analyses and modifications to the analyses revised by them before, so that they could agree more with the results, knowing that these results are reliable and well described.**

Reviewer's Responses to Questions

**Key Review Criteria Required for Acceptance?**

As you describe the new analyses required for acceptance, please consider the following:

**Methods**


-Are the objectives of the study clearly articulated with a clear testable hypothesis stated?

-Is the study design appropriate to address the stated objectives?

-Is the population clearly described and appropriate for the hypothesis being tested?

-Is the sample size sufficient to ensure adequate power to address the hypothesis being tested?

-Were correct statistical analysis used to support conclusions?

-Are there concerns about ethical or regulatory requirements being met?


Reviewer #1: "... Transportation Efficiency Index (TEI)[12], with values between zero and one, where values near to one indicate provinces better communicated, and considering such scale; we decided to standardize the TEI." The justification and the methods used are both rather unclear here.

**Thank you for your valuable observation. In few words, the TEI is constructed through Data Evelopment Analysis, an application of linear programming, allowing to compare decision making units, in this case the provinces, by optimizing a linear function consisting of outputs subject to linear restrictions depending on the inputs and outputs. The inputs represent the communication infrastructure and the outputs are transportation related; thus, obtaining an indicator of the extent in which each province efficiently utilize their transportation infrastructures. We rewrote the description of the TEI to reflect better its definition, the standardization, and the reason for such standardization (L-118)**

"It is important to notice that, in order to obtain more accurate rates of cases with COVID-19, population size in 2020 by province was linearly extrapolated using the corresponding information in the population and housing censuses from 2011 and 2016." It is not my field, but I am guessing that the demographers have techniques for anticipating "future" populations that would be more robust than simple linear extrapolation.

**Thanks for your commentary. Linear extrapolation in the demography framework corresponds to a mathematical projection method, the arithmetic increase one. We have projected and compared the population by using other mathematical methods: geometric, exponential, and logistic or saturation method and compared with those obtained by the United Nations** **through probabilistic projections** [**https://population.un.org/wpp/Download/Probabilistic/Population/**](https://population.un.org/wpp/Download/Probabilistic/Population/)**. Results associated with these projections are not provided by province by the UN, only for all Iran. However, we observed that once aggregated by country, the projected values we obtained by province are close to the UN projection value, particularly for those projections obtained through the geometric or exponential methods. We performed the spatial analyses with these different projections , and even using only the information of 2016 (which was discussed in the previous version of the manuscript) obtaining similar results in all cases in terms of the raw, smoothed rates, excess risk, and all the maps (the spatial lag model does not depend on these population projections). The results are attached in an Excel file (Supplementary Table ) .**

**Thus, we think that the projection is not as relevant and the effect of a sub-estimation of near to 34,000 individuals that we obtained when compared with the UN method through the geometric method distributed between 31 provinces, each having more than 700,00 individuals, seems to be negligible. Particularly, comparing the lineal extrapolation (arithmetic method), since it is the method we presented in the manuscript, with the geometric method, which has the estimations closer to that of the UN, for instance we observe a maximum difference between estimations of smoothed rates of 0.657 for every 100,000 people, corresponding to the province of Qom. That is, through the arithmetic method we obtain a rate of COVID19 cases of 76.1211 whereas with the geometric method a rate of 75.46392, both for every 100,000 individuals.**

**We obtained the maps associated with all methods. For simplicity, as an example we present here maps concerning the geometric vs arithmetic methods for the excess risk, smoothed rates, and clusters. We observed that the groups and clustering are quite similar among the different types of projections.**

**Geometric projection	Arithmetic projection (linear extrapolation)**

**We added information on this matter in the Discussion section (L- 353 and L-128)**

Reviewer #2: Details in paragraph starting on L62 seem more appropriate for the discussion than an introduction for the present analysis (e.g., chronic disease, epidemiological transition). I suspect the authors intended to introduce the importance of sociodemographic factors for infectious diseases because they examine some in their analysis. A general introduction to each one (or major themes) would be preferable to explain why these sociodemographic variables were chosen among all others.

**Thanks for your valuable suggestion we have added discussion on sociodemographic variables in the revised version of the manuscript.**

The authors highlight spatial analyses have been applied to previous infectious diseases but only cite one investigation of malaria (Chipeta et al., 2019). More citations are necessary to support the authors’ claim.

**Thanks for your suggestion, we have added to the paper other studies:**

1. **Lin D, Cui Z, Chongsuvivatwong V, et al. The geno-spatio analysis of Mycobacterium tuberculosis complex in hot and cold spots of Guangxi, China. *BMC Infect Dis*. 2020;20(1):462. Published 2020 Jul 1. doi:10.1186/s12879-020-05189-y**
2. **Deutsch-Feldman M, Brazeau NF, Parr JB, et al. Spatial and epidemiological drivers of *Plasmodium falciparum* malaria among adults in the Democratic Republic of the Congo. *BMJ Glob Health*. 2020;5(6):e002316.**
3. **Sanna M, Wu J, Zhu Y, Yang Z, Lu J, Hsieh YH. Spatial and Temporal Characteristics of 2014 Dengue Outbreak in Guangdong, China. *Sci Rep*. 2018;8(1):2344. Published 2018 Feb 5. doi:10.1038/s41598-018-19168-6**
4. **Carmo RF, Nunes BEBR, Machado MF, Armstrong AC, Souza CDF. Expansion of COVID-19 within Brazil: the importance of highways [published online ahead of print, 2020 Jun 27]. *J Travel Med*. 2020;taaa106. doi:10.1093/jtm/taaa106**

The aim of the paper in the sentence on L78 appears hidden among the statements of broader impact. The sentence also contains detail previously shared in the same paragraph. Clearing up the final sentence would end the Introduction on a strong foundation.

**Thanks for the commentary, this paragraph (L-87) was modified to reflect this comment.**

The authors declare the data came “mainly” from two sources, which suggests there are other sources. What were the other data sources?

**The data came from three sources, the Statistical Centre of Iran, Iran Data portal, and the Transportation Efficiency Index came from a paper. We have rewritten this part (L-104) to clarify which data come from which source.**

Variables were used raw or calculated, but it is not clear which variables fell under those two categories or how the variables were calculated.

**We have rewritten this section to make it clear which variables were already available and which ones were calculated (same paragraph as in the previous comment).**

The authors made efforts to extrapolate population levels of each province to more contemporary levels but did not for sociodemographic or climate variables, why not? If outside the scope of this paper, the authors can include a discussion of the potential limitations in the data availability.

**Thanks for your suggestion, a sentence concerning this limitation was added to the manuscript (L- 347). Formally, all variables should have been extrapolated; however, this process is quite complicated and out of the scope of the present manuscript since it involves accumulated information for several years which in many cases is unavailable or very difficult to obtain. Additionally, some explanatory variables might have a lagged in time effect over the response.**

The authors are missing citation(s) for why a linear extrapolation for population was used. Instead of extrapolating, authors could consider using predicted data from global models such as WorldPop (doi: 10.5258/SOTON/WP00645) and spatially aggregating by province.

**Thanks for the commentary, we already had included in the limitations part of the Discussion section that we used both the 2016 information and extrapolated information for 2020 obtaining similar results. However, to be sure that the projection method was irrelevant when analyzing the COVID-19 rates, we have employed additional extrapolation processes common in Demography obtaining similar results. Please refer to the answer provided to the second comment of Reviewer 1.**

How was TEI standardized?

**Please refer to the answer provided for the first comment of Reviewer 1. Briefly, it was used the value of each province minus the mean associated with all provinces and divided by the standard deviation.**

The average temperature of provincial capital cities and annual precipitation levels in 2015 are climactic variables and seem out of place among the sociodemographic factors in the analysis. There was no reference to this type of variable in the Introduction. The choice of a single temperature of the capital city of each province appears arbitrary without supporting detail from the authors. Were annual precipitation levels also from the capital cities? If a single metric is desired, an average temperature or total precipitation across an entire province would be more representative of climate than a single city.

**We included such variables since there was the hypothesis that greater temperature was associated with less cases of the disease, and similar for precipitation. Moreover, it was thought that CoVs survive longer in the environment at lower temperatures and lower relative humidity. A reference concerning this matter is for instance**

Aboubakr HA, Sharafeldin TA, Goyal SM. Stability of SARS-CoV-2 and other coronaviruses in the environment and on common touch surfaces and the influence of climatic conditions: a review [published online ahead of print, 2020 Jun 30]. *Transbound Emerg Dis*. 2020;10.1111/tbed.13707. doi:10.1111/tbed.13707.

**Though we know that climate information associated with all province is preferable than information of only the capital, we were not able to find the former. This information is provided by I. R. I Meteorological organization and available in the Statistical Centre of Iran. Unfortunately, we do not know whether the reason for presenting the information in this way is that it is not available in English or that the reliable meteorological devices are only in the capital of the provinces. We added this matter as a limitation of our study in the Discussion (L- 315).**Final del formulario

Suggestion to convert the number of physicians and hospital beds to a rate to account for population levels.

**We applied this suggestion, calculating the number of physicians and hospital beds for every 10,000 people in 2006. However, we obtained in general a worst fit of the model. For instance, the Lagrange Multiplier statistic to determine whether the spatial lag model fits the data is 3.7604 with p-value of 0.05248 (before it was LM=8.1953, p-value=0.004), and the other type of model, spatial error model, does not fit as well (LM=0.0988, p-value=0.75324). Additionally, the determination coefficient is 0.742 now instead of 0.87 and these variables are not significant. Based on those observations, we retained non-standardized numbers for those models.**

Suggestion to use “cumulative” cases instead of “total number” of cases and how were cases confirmed? What is the official Iranian definition?

**Thank you, we have changed to “cumulative” instead of “total”, as it is more precise for the definition, the Iranian official definition is the same as the WHO (**https://www.who.int/)

For the spatial linear models, the authors examined log-transformed cumulative case frequency instead of rates without explanation, why?

**We added a sentence concerning this matter (L- 167). There were two main reasons for using the transformed cumulative cases:**

**First, we have already discussed the population in 2020 is a projection, thus the rates might not be exact, and we preferred to use for the model non-projected information. The second, and most relevant, is that considering the dependent variable as counts, the normal distribution assumption under a logarithmic transformation associated with the model was satisfied, which was not true when we fitted the model using rates. Of course, we could have changed the model to consider a Poisson distribution, for instance by using a generalized geographically weighted regression, but we thought that analyzing the number of cases was as well relevant and that a model based on spatial weights was relevant since the results can be compared with those obtained with the spatial clustering and autocorrelation, methods also based in spatial weighting.**

The authors did not assess possible multicollinearity of the sociodemographic (and climate) variables before conducting their spatial linear models. It is also not clear in Methods if the authors conducted univariate spatial linear models or multivariate spatial linear models.

**Thanks for your observation, we did not assess multicollinearity for our final model. We have calculated association between the explanatory variables through a Kendall coefficient since variables are of an ordinal scale. We obtained the associations represented in the following figure:**

**Thus, we identified that variable “hospital beds” is strongly associated (with a correlation coefficient above 0.55) with GDP and with Physicians. Thus, we decided to eliminate it from the model. We now explain this in the Results section (L-199) and we have recalculated the parameter estimates and other statistics to reflect this change in all the results section and Table 2.**

**The spatial model was multivariate, we have added this in the name of the section as well as in the explanation in the results section (L-165). In fact, in the results we had written that all interpretations “consider fixed values for all variables except the one being interpreted”, which we thought made clear that the model was multivariate.**

Reviewer #3: Need a bit of clarification of the methods.

**Thanks for your suggestion following your observations and from the other reviewers, we have improved this section by clarifying some aspects that all reviewers kindly pointed out, including the Data section.**

--------------------

**Results**


-Does the analysis presented match the analysis plan?

-Are the results clearly and completely presented?

-Are the figures (Tables, Images) of sufficient quality for clarity?


Reviewer #1: (No Response)

Reviewer #2: General: Keep a consistent font, spacing, and number of significant figures for reported stats. Also, consider covering “multiplicative effects” to a percent increase.

**Thanks for your suggestion we have improved the spacing and number of figures.**

**As suggested, we reported all results concerning the spatial lag model in terms of percentage increase or decrease.**

The Results contain a large amount of results interpretation especially in subsections entitled “Predictors of COVID-19 spatial spread in Iranian provinces” and “Spatial lag predictors and province clusters.” All interpretations should be moved to the Discussion.

**We have moved from this section several paragraphs to the discussion and methods (mainly from L-176, from L- 277, paragraph from L-315)**

The sentence on L152 reads awkwardly.

**The sentence was re-written (L-184)**

I see a light blue color rather than purple in Figure 2A.

**It is correct, that color represents the Low-High cluster, and it is the same used in all figures. Maybe the problem is that it is more a light purple, we have added such adjective to the colour in the text.**

The syntax L167-168 suggests an interpretation of results and not a reporting of results.

**It is not an interpretation of results. We think it is necessary to present here how the spatial lag model fitted to the data, having a good fit, and to compare with the fit of the spatial error model, which did not fit well, both models being possible candidates. Thus, the latter model was not fitted or analyzed. We have included the statistics associated to the fit of the spatial error model (Lagrange multipliers), so that the reader can verify that it does not fit well. We have rewritten this part to reflect this process (L-205).**

Details on L188-190 are methodological and should be moved to the Methods.

**We were trying to point out which variables were significant. We have rewritten the sentence to clear this intention and included the p-value associated to these significant variables (L-213).**

Consider using a different word for “confirmed” on L199 with a neutral verb.

**Thank you, the word was changed.**

The global spatial autocorrelation between cases and temperature is insignificant by your specified threshold (L204), and should be interpreted cautiously.

**Since we moved this part to the discussion, we pointed out this matter on such section (L-317).**

Consider removing “as expected” on L213 from Results.

**We have now made this change**

Reviewer #3: Yes

--------------------

**Conclusions**


-Are the conclusions supported by the data presented?

-Are the limitations of analysis clearly described?

-Do the authors discuss how these data can be helpful to advance our understanding of the topic under study?

-Is public health relevance addressed?


Reviewer #1: (No Response)

Reviewer #2: The section could start on a stronger foundation if the title sentence ended before the clause “probably due to...” and save the second clause as a possible explanation of the spatial heterogeneity (with supporting citations).

**We modified this sentence as suggested.**

The authors’ focus on the sociodemographic findings and their broader impact (as well as using words such as “determinants” or “leads to”) should be tempered because their choice in methodology was exploratory. For example, bivariate analyses are useful to explore the relationship between case rate and a spatial variable, but they do not control the correlation between the two at each location and should be interpreted with caution. A discussion of this limitation is missing and can be combined with L181-185 in the Discussion.

**The statement of the correlation between each location was already stated in the Results (L181-185) and maybe was too hidden. We have moved it to the discussion and completed the discussion about this matter as suggested (L-321). However, most of our interpretations were in terms of the spatial multivariate linear model and not in terms of the bivariate** **analyses. We also now stress along all document that we are deriving associations and not causality.**

Even though the authors introduced the possibility of underreporting of COVID-19 cases in Iran in the Introduction (L60-61), the authors do not discuss reporting as a limitation in their paper. Regardless of the reliability of data reported in the Statistics Center of Iran, the degree of testing or availability of testing and their possible spatial heterogeneity was not accounted for or discussed in the paper as a limitation to their analysis.

**Thank you, we have added this part in the discussion an discussed this limitation with regards to testing.**

Using “protective effect” suggests causation and should be reworded in the context of this exploratory analysis. Also, recommending public health action based on univariate associations should be provided with caution because possible confounding by other factors has not been accounted for in the present analyses.

**The word protective and all words meaning causation were removed from all the document. From this sentence, we infer that the reviewer thought the spatial linear model was applied separately for each explanatory variable, however, the model is multivariate and has been referred as such in the revised version of the manuscript.**

Also missing is a discussion of the similarity in raw and smoothed rates as well as a discussion of the differences in the spatially lagged and spatial error models.

**We added in the results the statistics associated with the spatial error model to clarify that it does not fit the data (please see answer to Reviewer 2 in the Results section above for “The syntax L167-168 suggests an interpretation of results and not a reporting of results.”). Hence, we think that perhaps it is not necessary to discuss more about the two different models. We have added to the discussion a sentence concerning the similarity between raw and smoothed rates (L-357).**

The strengths of the study (L276) focus on the broader impacts and do not comment on the strength of the methodology and overall approach.

**Thank you. We have added several sentences concerning the strength of our methodology (L-335).**

The authors did not discuss potential limitations of their climate analysis. See details in previous comment in Methods.

**Please, refer to the answer of the same reviewer (reviewer 2). We have added a paragraph (L-315) concerning climate in the discussion.**

On L287, I suspect the authors mean using projected and reported “population” not cases. Not clear how this supports study “feasibility” but rather robustness of their extrapolation approach.

**Thank you for your observation, it is correct.**

In addition to a future direction of using spatio-temporal analyses, the authors could consider a multivariate spatial regression to account for some of the limitations in their present analysis.

**The model is already multivariate as we have clarified in the manuscript, bivariate analyses were performed for the significant variables in this multivariate spatial model to have a closer study of the spatial relationships. Both aspects are more stressed now.**

Reviewer #3: The discussion section needs to be rewritten to avoid language confusion correlation with causality.

**We modified all the discussion and paper to clarify that we are talking about association and not causation.**

--------------------

**Editorial and Data Presentation Modifications?**

Use this section for editorial suggestions as well as relatively minor modifications of existing data that would enhance clarity. If the only modifications needed are minor and/or editorial, you may wish to recommend “Minor Revision” or “Accept”.

Reviewer #1: "Iran has one of the highest COVID-19 mortality rates fluctuating between 8 to 18 percent daily, and..." This statement is quite unclear ... 8-18% of the individuals infected, or what?

**We agree that this statement might have been confusing and have since removed it from the manuscript.**

"The large count or COVID-19 cases..." should this be "of"?

**It was a typo, thank you.**

"... that improves variance instability proper of rates estimated in small-sized spatial units..." I cannot understand this phrase.

**We mean that** **provinces with a larger population size have a lower variance than provinces with a smaller population size. This is a statistical property inherent to proportions.** **We have added a small parenthesis to clarify this idea to the reader (L-137).**

Reviewer #2: Tables/Figures:
T1: An indication of which variables were calculated and the source of all data would improve the table.

**We have added in both the text and table the source each data came from.**

General: Colorkey titles should be descriptive instead of raw variable name. Translate “conteo” to “count”.

**We have now made these changes.**

F1B: The highest level in the colorkey has 0 provinces. Consider removing and reorganizing the colors.

**We have now made these changes.**

F2: Even though the colors are geoDa defaults, consider custom colors to help with colorblind readers.

**We have reduced saturation for all images to make them more colorblind friendly. We hope these modifications are useful.**

Mechanics:
General typo “SARS-COV2” is “SARS-CoV-2”

**Corrected**

L62 typo

**Corrected**

Abstract:
The authors should report more specific details from their study including: 1) the time frame of the study, 2) the name of the spatial analysis techniques, 3) statistical results (e.g., effect size, p-value) from their analysis, 4) at least one limitation or caveat to their analysis.

**The abstract was modified to include all these four suggestions**

Reviewer #3: Some wording, highlighted in my review

Review of PNTD-D-20-00725: “Spatial analysis of COVID-19 spread in Iran: Insights into geographical and structural transmission determinants at a province level”

This manuscript is a regression analysis of COVID-19 patterns in Iran, performed at a provincial level, and taking into account the spatial structure of the variables used. As such, I found it interesting and well performed (however, I am no expert in public health). I have a few minor comments, but a more substantial one: this is a correlative study, but in the discussion, the language too often suggests an understanding of causation for which there is no evidence. Although this needs to be corrected, it is easy to rewrite the discussion using more careful wording.

**Thank you, the document, and in particular the discussion, was revised to avoid words related to causality instead of association.**

Some details.

Lines 64-65. What is the meaning of “…its economic situation … is among the highest”? A situation cannot be high or low. Please rewrite.

**The sentence (L-78) was properly rewritten considering this comment.**

Line 97. How was the data “standardized”? There are many ways of standardizing a variable. Be explicit.

**We added in the document the standardization process, which was the usual, the TEI minus its mean divided by the standard deviation, to allow interpretations in terms of the increase of a certain number of standard deviations since the interpretation in terms of the raw values was confusing. Please refer to the answer provided to Reviewer 1, first comment, in Methods.**

Line 120. I believe they actually performed bootstrapping on the data, rather than “…Monte Carlo simulations”, for which the Moran paper cited is NOT a right reference. Correct this and be careful with the references.

**The reference was misplaced, it corresponded to Moran’s I, not to the inference associated with the statistic, so that it was moved accordingly. We have added a reference concerning the simulations and corrected the name of the method and explanation (L-152), which effectively is not referred by Luc Anselin (programmer of GeoDa) as Monte Carlo simulations. In words of Luc Anselin: “Inference for Moran’s I is based on a null hypothesis of spatial randomness. An alternative to an analytical derivation is a computational approach based on *permutation*. This calculates a reference distribution for the statistic under the null hypothesis of spatial randomness by randomly permuting the observed values over the locations. The statistic is computed for each of these randomly reshuffled data sets, which yields a *reference distribution*.” Thus, it seems that strictly the method is also not a bootstrap method, though close.**

Lines 136-137. A reference is required here. It is not clear to every reader what a “Lagrange Multiplier statistic” is.

**The Lagrange Multiplier or score test (which have been proved to be equivalent) has been mainly used in econometric analyses to contrast a hypothesis concerning restricted parameters, in our case to see whether the spatial parameter rho is equal to zero. Its name comes from the underlying process to derive it, which corresponds to maximizing a score function (the gradient associated with the log-likelihood) subject to restrictions, which in calculus involves Lagrange multipliers. In simple words, and in our case, through this test, we see how much improvement there is in the slope of the likelihood when the spatial parameter is included. A reference was added corresponding to Lagrange multipliers.**

Lines 234-235. You just showed statistical associations. Whether this shows or not what is needed to “model the disease adequately” is a different matter. The discussion needs a more careful language. For instance, in Line 237, what would be the role of variables in a mechanistic model is not established. Or (Line 265), that literacy is a “protective effect.” You just showed that some variables covary, not that one factors “protects” another.

**The reviewer is correct. All the document was revised, especially the Discussion, to avoid words concerning causation.**

--------------------

**Summary and General Comments**


Use this section to provide overall comments, discuss strengths/weaknesses of the study, novelty, significance, general execution and scholarship. You may also include additional comments for the author, including concerns about dual publication, research ethics, or publication ethics. If requesting major revision, please articulate the new experiments that are needed.

Reviewer #1: The statement "Spatial statistics have emerged as a novel multidisciplinary tool for the analysis of spatial epidemiology ..." is a bit overblown. That is, spatial statistics have been around for decades, and has been the basis for spatial epidemiology. I don't see the "novel" there, although there are certainly novel spatial statistics that could be applied to these questions. I am just wanting to see no overblown statements.

More generally, I think that this manuscript presents interesting results, although perhaps it is a bit of using complex spatial statistics to show something that is more or less obvious ... COVID-19 cases in the north, and fewer in the south. I am not sure that there is a deep and profound impact of these results on the understanding of this outbreak.

**Thank you for your comments, we have modified the paragraph including that statement in order to not imply that spatial statistics is a recently introduced methodology.**

**We think that the results we obtained perhaps confirm aspects that are more or less known, for instance the association of old age with the disease; however, we are able to see the strength of this association through the exponentiated parameters corresponding to the multivariate spatial linear model. And, by adding a spatial effect, we consider a basic geographical principle stating that what occurs in a place is correlated with what happens in near places. Additionally, we think we were also able to see how the disease behaves geographically by its own, in terms of rates and significant clustering, and when combined with other variables. In this sense, we tried to include variables that were suspected or known to be associated with the number of cases; thus, we think, that maybe even non-significant variables provide information about which factors might not be associated with the disease.**

Reviewer #2: The authors provide a spatial analysis of a timely topic of global proportion (COVID-19) in a country with high mortality outside of China in the early phase of the pandemic (Iran). The paper is relevant to global health and examines COVID-19 in a resource-poor nation and is, therefore, relevant to the journal. The authors suggest the largest contribution of their paper is to inform policy, instead it is my opinion the impact of this paper is methodological whereby on-going COVID-19 models must account for the spatial autocorrelation in cases, as the authors mention briefly. The ecological study attempts to identify social factors related to case rates, which are appropriately stabilized using spatial smoothing methodology. However, there are major concerns that affect the design and clarity of the study that should be considered before publication.

**Thank you, we have stressed more the methodology and its relevance according to your suggestions. We also think that with all the modifications, we have clarified aspects related to the methodology and in general of all the study greatly improving the manuscript.**

Reviewer #3: NA

**Do you want your identity to be public for this peer review?** For information about this choice, including consent withdrawal, please see our [Privacy Policy](https://www.plos.org/privacy-policy).

Reviewer #1: No

Reviewer #2: Yes: Ian Buller, Ph.D., M.A.

Reviewer #3: No
