# Supplementary material for: Spatial analysis of COVID-19 spread in Iran: Insights into geographical and structural transmission determinants at a province level
Source: PLoS Negl Trop Dis. 2020 Nov 18;14(11):e0008875. doi: 10.1371/journal.pntd.0008875 (PMC7710062; doi:10.1371/journal.pntd.0008875)
Supplement: Attachment — Submitted filename: Answer_Reviewers_2nd_07092020.docx [file pntd.0008875.s004.docx]

September 7^th^, 2020

**Dr. Townsend Peterson**

**Guest Editor**

**PLOS Neglected Tropical Diseases**

On behalf of my co-authors and myself I am re-submitting the manuscript entitled “Spatial analysis of COVID-19 spread in Iran: Insights into geographical and structural transmission determinants at a province level”. We send the corrected manuscript and the same manuscript with changes highlighted. At the bottom of this letter we include the comments by the reviewers along with our response, with the location of the changes when appropriate.

We appreciate the opportunity of considering our work,

Sincerely yours,

Ricardo Ramírez-Aldana, Juan Carlos Gomez-Verjan, Omar Yaxmehen Bello-Chavolla

Correspondence

Ricardo Ramírez-Aldana

Research Division

Instituto Nacional de Geriatría

Periférico Sur No. 2767

Col. San Jerónimo Lídice,

Del. La Magdalena Contreras, D.F. 10200

ricardoramirezaldana@gmail.com

From Associate Editor Town Peterson:

It appears that the authors conducted a rather profound revision, and my reviewer, who was previously critical, is now mostly happy. They do point out, and I agree, that an analysis that includes a model selection step would be important--so I am saying "minor revision," but I would like to see this analysis added before I do a final acceptance. So please work through the reviewer's comments, and pay special attention to the model selection part?

All the best, ATP

**Thank you for noticing all the work we performed to improve the manuscript. The model selection strategy was performed and the other minor concerns were attended and the manuscript was modified considering all the suggestions and recommendations.**

Reviewer's Responses to Questions

Key Review Criteria Required for Acceptance?
As you describe the new analyses required for acceptance, please consider the following:

Methods
-Are the objectives of the study clearly articulated with a clear testable hypothesis stated?
-Is the study design appropriate to address the stated objectives?
-Is the population clearly described and appropriate for the hypothesis being tested?
-Is the sample size sufficient to ensure adequate power to address the hypothesis being tested?
-Were correct statistical analysis used to support conclusions?
-Are there concerns about ethical or regulatory requirements being met?

Reviewer #2: After the previous round of review, the authors included a test for multicollinearity and reduced the variables used in their spatial multivariate linear models. However, the present study uses all available explanatory variables simultaneously in a model without a model selection scheme, which may increase the chance their models are overfitting the data. The objective of this part of the present analysis is “to identify variables that significantly impact the number of log-transformed COVID-19 cases” performing model selection will help achieve their goal.

**To answer this concern, we used a Backward selection method. Considering a significance level of 0.1 and without considering any information criterion in the selection process, we obtained in a fourth step, a model without population density, Consumer Price Index, and annual precipitation. We obtained the same significant variables as those obtained in the model including all variables: percentage of people settled in urban areas (p-value = 0.008), smoothed rate of people aged ≥60 years (p-value < 0.001), literacy rate (p-value = 0.010), average temperature (p-value < 0.001), number of physicians employed (p-value < 0.001), and the TEI (p-value = 0.037). The estimated coefficients were almost the same as the ones obtained when fitting a model including all variables. The goodness of fit measures were also similar: coefficient of determination of 0.87 now vs 0.88 before, SE of regression of 0.39 now vs 0.38 before; and the Akaike Information Criterion (AIC) was improved, decreasing from 57.165 to 52.705.**

**If we considered a Backward selection method with a lower significance level of 0.05 and without considering any information criterion, apart from the variables eliminated before, we would eliminate GDP and TEI at the last sixth step (TEI having an associated p-value of 0.059). However, all goodness-of-fit measures were worsened when compared with both the model including all variables and the simplified model obtained above: coefficient of determination of 0.84, AIC of 54.783, and SE of regression of 0.437. In fact, when considering every step of the selection process the model obtained in four steps provides the smallest AIC, even comparing with the original model. Thus, the model obtained in four steps is the model in which the AIC is minimized. The AIC associated with each step is as follows:**

**First step: 57.1648, Second step: 55.230, Third step: 53.8472, Fourth step: 52.7049, Fifth step: 53.4121, Sixth step: 54.783**

**Considering the previous discussion, we decided to use for the selection process both the significance level (0.05) and minimization of the AIC as selection criteria, obtaining the model already discussed in the first paragraph of this answer. In this way, the variables with less associated significance are eliminated and a parsimonious model is considered. The values of the estimated coefficients, p-value, etc. associated with this model were added to Table 1 and the process we followed was added in both the methods and results sections and some lines were modified accordingly (L-185, L-192, L- 221, L- 234).**

In L148, the data agreement can be paraphrased and shortened, or removed.

**The sentence previous to this line was modified and the mentioned line was removed (L-146).**

In L368, the authors mention the possibility “that some variables have a time lagged effect over their response” but the authors do not test for temporal lag in their analysis. Why not? If it is because data was unavailable as mentioned in L367, then I recommend discussing this as a limitation of the study.

**This sentence corresponds to a possibility that, in certain way, we are considering in our models and maybe it was not properly explained. For instance, GDP might have a time lagged effect in the sense that in those regions with better conditions some years ago perhaps there is now a better infrastructure to support their population in terms of health and education. Thus, even though in 2020, the GDP in a hypothetical region is inferior, what was important was the economic situation of this region years ago. However, since we are using information which was publicly available, and not with the intention of properly analyze the time effect, we are not obtaining information that maybe according to experts has the correct time lagged effect. For instance, in this example of the GDP, perhaps the time lag should have been 5 years instead of 16 that we used.**

**We have included a sentence to clarify this issue and to discuss it as a possible limitation (L- 380).**

Results
-Does the analysis presented match the analysis plan?
-Are the results clearly and completely presented?
-Are the figures (Tables, Images) of sufficient quality for clarity?

Reviewer #2: Recommend sentence in L243 to be moved to discussion.

**As suggested by the reviewer this sentence was moved to the discussion section (L-300)**

Recommend adding the figure of the multicollinearity matrix to supplemental materials.

**The correlation matrix was added as part of the supplementary material (L- 222).**

Conclusions
-Are the conclusions supported by the data presented?
-Are the limitations of analysis clearly described?
-Do the authors discuss how these data can be helpful to advance our understanding of the topic under study?
-Is public health relevance addressed?

Reviewer #2: The relationship between climate and SARS-CoV-2 transmission is a contentious topic, one that requires more investigation. The present study lends mixed support to this relationship. And while the authors cite a review (43) and many studies within that review have found a similar relationship to one of the results within the present study, it would be important to note contrary findings. Such as, for example, Jamil et al. 2020 ([https://www.medrxiv.org/content/10.1101/2020.03.29.20046706v2](https://www.medrxiv.org/content/10.1101/2020.03.29.20046706v2)) who is also cited and discussed within (43), especially in light of the present study climate data limitations.

**A sentence was added considering this issue and the reference was added (L-353).**

Recommend replacing “though with a p-value of 0.103” with “not statistically significant” or something similar to help the reader interpret the results.

**The suggestion was attended and the line was modified (L-344)**


Editorial and Data Presentation Modifications?
Use this section for editorial suggestions as well as relatively minor modifications of existing data that would enhance clarity. If the only modifications needed are minor and/or editorial, you may wish to recommend “Minor Revision” or “Accept”.

Reviewer #2: There are grammatical errors throughout the newly added sections (highlighted in yellow), I recommend light copyediting.

**All the paper was revised, in particular all sections highlighted in yellow, to identify grammatical errors or typos and modified accordingly.**

Summary and General Comments
Use this section to provide overall comments, discuss strengths/weaknesses of the study, novelty, significance, general execution and scholarship. You may also include additional comments for the author, including concerns about dual publication, research ethics, or publication ethics. If requesting major revision, please articulate the new experiments that are needed.

Reviewer #2: The authors return with a greatly improved manuscript of their study in a country affected greatly early in a global pandemic. The authors responded thoroughly, thoughtfully, and professionally to the previous reviewers, evident especially in the improvements to the tables and figures. The methods are clearer and their limitations are more encompassing than the initial submission. A few methodological concerns remain regarding the investigation into variables that are associated with cases as well as caution for the interpretation of the relationship between climatic variables and cases.

**Thank you for all your suggestions which have greatly improved the paper. The two methodological concerns were attended and the manuscript was modified accordingly.**
